# A prospective, non-randomized phase II trial of Trastuzumab and Capecitabine in patients with HER2 expressing metastasized pancreatic cancer

**DOI:** 10.1186/1471-2482-9-1

**Published:** 2009-01-08

**Authors:** Andre Mihaljevic, Peter Büchler, Jan Harder, Ralf Hofheinz, Michael Gregor, Stephan Kanzler, Wolff Schmiegel, Volker Heinemann, Esther Endlicher, Günter Klöppel, Thomas Seufferlein, Michael Geissler

**Affiliations:** 1 Department of General Surgery, University of Heidelberg, Im Neuenheimer Feld 110, 69120 Heidelberg and Klinikum rechts der Isar, Technische Universität Munich, Ismaninger Strasse 22, 81675 Munich, Germany; 2 Department of Gastroenterology and Hepatology, Freiburg University Hospital, Hugstetterstr, 55, 79106 Freiburg, Germany; 3 Department of Internal Medicine I, University Clinic Tubingen, Ottfried-Müller-Str. 10, 72076 Tübingen, Germany; 4 Department of Internal Medicine I, Johannes Gutenberg University, Langenbeckstr, 1, 55101 Mainz, Germany; 5 III. Medical Clinic, University Hospital Mannheim, University of Heidelberg, Wiesbadener Strasse 7-11, 68167 Mannheim, Germany; 6 Department of Medicine, Ruhr University Bochum (Knappschaftskrankenhaus), In der Schornau 23-25, 44892 Bochum, Germany; 7 University of Munich, Medical Department III, Klinikum Muenchen-Grosshadern, Marchioninistrasse 15, 81377 Munich, Germany; 8 Department of Internal Medicine I, University of Regensburg, Franz-Josef-Strauß-Allee, 93042 Regensburg, Germany; 9 Department of Pathology, University of Kiel, Michaelisstr.11, 24105 Kiel, Germany; 10 Department of Gastroenterology, University of Ulm, Robert-Koch-Str. 8, 89081 Ulm, Germany; 11 Department of Medicine, Gastroenterology and Oncology, Municipal Hospital Esslingen, Germany

## Abstract

**Background:**

Pancreatic cancer is the fourth most common cause of cancer related death in Western countries. Advantages in surgical techniques, radiation and chemotherapy had almost no impact on the long term survival of affected patients. Therefore, the need for better treatment strategies is urgent. HER2, a receptor tyrosine kinase of the EGFR family, involved in signal transduction pathways leading to cell growth and differentiation is overexpressed in a number of cancers, including breast and pancreatic cancer. While in breast cancer HER2 has already been successfully used as a treatment target, there are only limited data evaluating the effects of inhibiting HER2 tyrosine kinases in patients with pancreatic cancer.

**Methods:**

Here we report the design of a prospective, non-randomized multi-centered Phase II clinical study evaluating the effects of the Fluoropyrimidine-carbamate Capecitabine (Xeloda ^®^) and the monoclonal anti-HER2 antibody Trastuzumab (Herceptin^®^) in patients with non-resectable, HER2 overexpressing pancreatic cancer. Patients eligible for the study will receive Trastuzumab infusions on day 1, 8 and 15 concomitant to the oral intake of Capecitabine from day 1 to day 14 of each three week cylce. Cycles will be repeated until tumor progression. A total of 37 patients will be enrolled with an interim analysis after 23 patients.

**Discussion:**

Primary end point of the study is to determine the progression free survival after 12 weeks of bimodal treatment with the chemotherapeutic agent Capecitabine and the anti-HER2 antibody Trastuzumab. Secondary end points include patient's survival, toxicity analysis, quality of life, the correlation of HER2 overexpression and clinical response to Trastuzumab treatment and, finally, the correlation of CA19-9 plasma levels and progression free intervals.

## Background

Pancreatic cancer it is the fourth most common cause of cancer related death in Western countries [1]. A recent database study revealed that surgical therapy is the most important predictor of long-term survival but only 10.4% of patients were resectable [2]. Median survival after resection was 13 months among compared to a median survival of 4 months in patients not amendable to surgery [2]. Since most patients develop tumor recurrence the mainstay of treatment is gemcitabine based chemotherapy [3,4]. Gemcitabine-based combination therapies did not lead to further improvements in survival, although the combination of gemcitabine with capecitabine has shown promising results [4]. A potentially promising combination therapy is capecitabine (Xeloda™) which can be orally administered and the molecular target compound Trastuzumab (Herceptin™), which is a neutralizing humanized antibody directed against the extracellular domain of the HER2 tyrosine receptor kinase.

Capecitabine is an orally applied fluoropyrimidine, which is converted into active 5-FU. Within this enzymatic cascade thymidinephosphorylase finally catalyzes the generation of active 5-Fluoruracil (5-FU). Thymidinephosphorylase is almost exclusively present in tumor cells, the activation of capecitabine to 5-FU is restricted to malignant cells. When applied twice daily capecitabine shows similar pharmacokinetics as a continuous 5-FU infusion [5]. Based on several clinical trials the toxicity of capecitabine appears low [6,7]. Common side effects include abdominal symptoms like diarrhea, nausea, vomiting as well as dermatologic symptoms like dermatitis or the hand-foot syndrome. Noteworthy, capecitabine has been reported to be cardiotoxic inducing myocardiac infarction, angina and arrhythmias [8]. Recent phase III trials comparing gemcitabine monotherapy to a combination of gemcitabine plus capecitabine did not result in a significant survival benefit [9-11].

Trastuzumab is a neutralizing humanized antibody directed against the extracellular domain of the HER2 tyrosine receptor kinase. Expression of the HER2neu oncogen is analyzed by immunohistochemistry (IHC) and fluorescent in-situ hybridization (FISH) in routine pathological specimen assessment. The antineoplastic effect of Trastuzumab is well documented in breast cancer and has been ascribed to cell cycle arrest and incuction of apoptosis as well as induction of antibody-dependent cellular cytotoxicity (ADCC) against HER2 – overexpressing tumor cells [12]. A number of promising pre-clinical studies in pancreatic cancer suggest a potential therapeutic benefit for those patients overexpressing HER2/neu [13-15]. The rationale for applying trastuzumab in patients with advanced pancreatic cancer is based on studies in which 11% of human specimens overexpressed HER2 with a score of 3 and 26% with a score of 2 [16,17]. In a recent phase II study in 21 patients with metastasized HER2 overexpressing (grade 2–3) pancreatic cancer a combination of trastuzumab and capecitabine resulted in partial remission rates of 24% and 50% of patients showed a reduction of the tumor marker CA19-9 [17].

Taken together, the need for novel therapeutic strategies such as target therapy in pancreatic cancer patients is obvious. One approach involves targeting the HER2/neu receptor in combination with cytotoxic agents. Based on the poor outcome of current therapies, we evaluate the activity and tolerability of the combination therapy of trastuzumab plus oral capcitabine in patients with proven HER2 overexpressing advanced pancreatic cancer.

## Methods

### Trial organization

This multicentric, multiinstitutional trial has been designed by the Centre of Clinical Trials, University Medical Center Freiburg and by LabConsult GmbH Freiburg. The trial medication (trastuzumab and capecitabine) is supplied by the Roche AG, Basel, Switzerland. Participating study centers are as follows:

• Department of General Surgery, University of Heidelberg, Im Neuenheimer Feld 110, 69120 Heidelberg, Germany

• Department of Gastroenterology and Hepatology, Freiburg University Hospital, Hugstetterstr. 55, 79106 Freiburg, Germany

• Department of Internal Medicine I, University Clinic Tubingen, Ottfried-Müller-Str. 10, 72076 Tübingen, Germany.

• Department of Internal Medicine I, Johannes Gutenberg University, Langenbeckstr. 1, 55101 Mainz, Germany

• III. Medical Clinic, University Hospital Mannheim, University of Heidelberg, Wiesbadener Strasse 7–11, 68167 Mannheim, Germany

• Department of Medicine, Ruhr University Bochum (Knappschaftskrankenhaus), In der Schornau 23–25, 44892 Bochum, Germany

• University of Munich, Medical Department III, Klinikum Muenchen-Grosshadern, Marchioninistrasse 15, 81377 Munich, Germany

• Department of Internal Medicine I, University of Regensburg, Franz-Josef-Strauß-Allee, 93042 Regensburg, Germany

• Department of Pathology, University of Kiel, Michaelisstr.11, 24105 Kiel, Germany.

• Department of Medicine, Gastroenterology and Oncology, Municipal Hospital Esslingen, Germany

### Coordination

The trial is co-ordinated by the Centre of Clinical Trials, University Medical Center Freiburg and by LabConsult GmbH Freiburg. This institution is responsible for overall trial management, trial registration, database management, quality assurance including monitoring, reporting and for the scientific program of all trial related meetings.

### Investigators

Patients will be recruited by all participating institutions. All investigators are experienced oncologists from the fields of medical oncology and general surgery at co-operating institutes.

### Adverse events management

For all severe serious adverse events the documentation and relevant patient data are verified by the co-ordinating personnel before submitting the data to the Center for Clinical Trials, Freiburg as well as to Roche (Roche Pharma AG, Grenzach-Wyhlen, Germany) with 24 h. The German authorities will be informed by Roche in the case of causal relationship. Severe adverse events are events that cause:

• death of study subject

• life threatening illness

• hospitalization or

• extend of hospitalization

• permanent or significant discomfort/disabilities

Patient toxicities will be assessed using the NCI Common Toxicity Criteria (CTC) 3.0 (June 2003). Toxicity will be evaluated before treatment, and prior to each course of trastuzumab and capecitabine and at follow-up. Unacceptable toxicity is defined as unpredictable, or irreversible Grade 4 toxicity.

### Medication supply

All chemotherapeutic agents are provided by the Roche (Roche Pharma AG, Grenzach-Wyhlen, Germany) upon request. Each request comprises 2 cycles (6 weeks).

### Quality control

Quality control is achieved by monitoring, auditing and data verification according to the good clinical practice guidelines (GCP). To ensure correct documentation a specially trained independent trial monitor will perform regular visits in the study centers as well as checks on the individual principal investigators. Source data verification is done by the monitor to ensure that all data present in the case report form corresponds to data in the original documents present in the patient folder. Finally audits will be held either by the sponsor or responsible authorities.

### Ethics, informed consent and safety

The final protocol was approved by the ethics committee of the University of Freiburg and by the local ethic committees of participating institutions. This study complies with the Helsinki Declaration in its recent German version, the Medical Association's professional code of conduct, the principles of Good Clinical Practice (GCP) guidelines and the Federal Data Protection Act. The trial will also be carried out in keeping with local legal and regulatory requirements. The medical secrecy and the Federal Data Protection Act will be followed. Written informed consent is obtained from each patient in oral and written form before inclusion and after nature, scope, and possible consequences of the trial have been explained by a physician. The investigator will not undertake any measures specifically required only for the clinical trial until valid consent has been obtained.

### Patient selection

**Figure 1:**
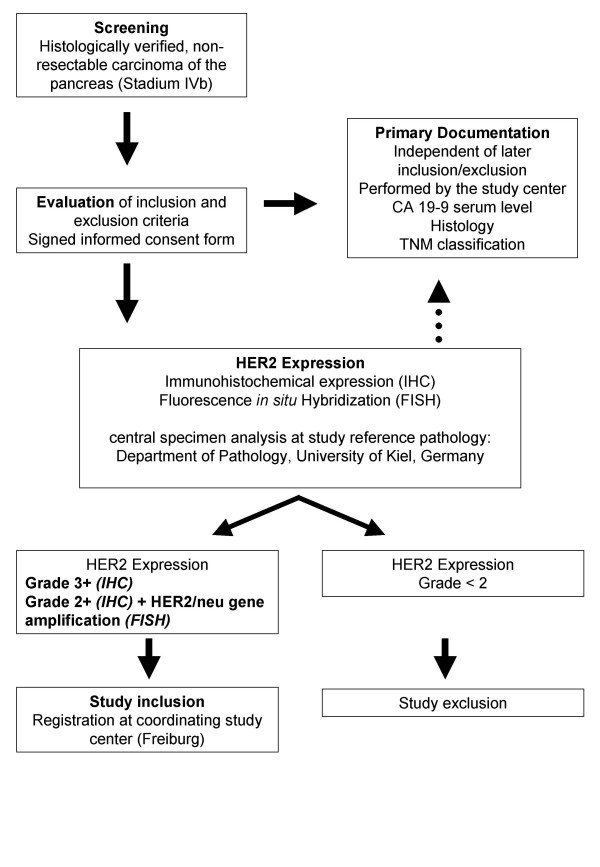
**Trial flow chart**. Flow chart summarizing the multi step process from initial patient contact until the inclusion of properly screened patient into the trial. Once diagnosis of pancreatic cancer stage IVb was established patients were screened for inclusion criteria. If patients met all inclusion criteria and no exclusion criteria prohibited participation in the study an informed consent was signed. All patients underwent histological confirmation and HER2 grading in the centralized reference pathology. Patients with HER2 grade 3 and patients with HER2 grade 2 plus proven HER2 gene amplification in FISH analyses were eligible for study inclusion. All other patients with HER2 grade 0, 1, or 2 without gene amplification were excluded from this study.

Patients over 18 years of age with advanced pancreatic cancer (Stage IVb) without the chance of surgical tumor removal are potential candidates for the study and will be screened for inclusion. Recruitment started during the first trimester of 2004 and is expected to be completed within a 36 months period. Table 1 outlines a detailed list of the eligibility criteria of this study. An outline of the recruitment algorithm is provided in Figure 1. A total of 37 patients will be included with an interim analysis held after 23 patients.

**Table 1:** List of the eligibility criteria

**Inclusion criteria**	**Exclusion criteria**
1. written informed consent	1. possible surgical resection and/or radiotherapy with curative potential
2. age 18 years or older	2. dihydropyrimidine-dehydrogenase deficiency
3. histological verified pancreatic cancer in stage IVB (T_1–4_N_0_M_1_)	3. gastrointestinal obstruction
4. staging and CA19-9 serum level not older than 4 weeks	4. a known secondary neoplasm except a curative treatable basalioma of the skin or carcinoma in situ of the cervix uteri
5. histological verified over-expression of HER2/neu (immunological score 3+ or 2+ and HER2/neu gene amplification in FISH analysis)	5. a known hypersensitivity against any of the applied substances
6. at least one measurable lesion (≥ 2 cm in conventional CT scan or ≥ 1 cm in spiral CT scans)	6. clinically relevant disorder of the cardio-vascular system or other organs or a severe systemic disease that compromises the study protocol or the interpretation of the data
7. no prior radiation or chemotherapy	7. clinically manifest pulmonary disorder
8. performance-status 0–2 according to WHO/ECOG or ≥ 60 points on the Karnofsky scale	8. prior polyneuropathy
9. life expectancy of at least 3 months	9. a concomitant treatment with the virusstatic agents Sorivudin or its analogues
10. Left ventricular excretion fraction > 50%	10. pregnancy, breast feeding or absence of appropriate contraceptive measures
11. appropriate renal, liver and hematopoetic function defined by:	11. psychiatric disorders, drug abuse or other disorders, that compromise the informed consent
a. neutrophils ≥ 1.5 × 10^9^/l	
b. hemoglobin ≥ 80 g/l	
c. platelets ≥ 100 × 10^9^/l	
d. total bilirubin < 3 × normal	
e. creatinine clearance ≥ 30 ml/min (Cockroft Gault)	
f. transaminases	
i. < 2.5 × normal	
ii. < 5 × normal in case of liver metastases	
12. possibility of long-term follow up	12. concomitant participation in other clinical trials or participation within the last 4 weeks
13. negative pregnancy testing	13. any other disorder or treatment that poses a risk to the patient or is incompatible with the aims of this study

### Study design and objectives

The study is designed as a prospective, open, one-armed multicentric Phase-II trial evaluating the efficacy of trastuzumab in combination with capecitabine compared to capecitabine alone in patients with advanced pancreatic cancer. Efficacy is evaluated by the primary study objective which is progression free survival after 12 weeks. Secondary study objectives are to assess efficacy by evaluating: a) progression free survival time; b) overall survival; c) time till remission (partial or complete); d) duration of remission; e) rate of "clinical benefit response" after 12 weeks; f) quality of life before treatment and after two 2 cycles of chemotherapy. Additional secondary trial endpoints include toxicity analysis, the rate of adverse events and the relationship between progression free survival and CA19-9 plasma levels. Finally, the relation between HER2/neu overexpression and progression free survival will be evaluated.

### Standardized treatment protocol

The trastuzumab loading dose of 4 mg/kg body weight will be given on day 1 over 90 min. For maintenance therapy a weekly dose of 2 mg/kg body weight over 30 min will be infused until tumor progression takes place. Patients will be monitored closely for 6 hours during the first dose of trastuzumab and for 2 hours after the following trastuzumab infusions to rule out adverse reactions. Capecitabine will be applied orally twice daily at a dose of 1250 mg/m^2 ^on day 1–14 followed by a break of 7 days. The three weeks cycles will be repeated till tumor progression or till a grade 3–4 toxicity occurs. A detailed overview over the treatment protocol is outlined in Table 2. In case of adverse reactions of capecitabine therapy, a modification of the dose will be performed (Table 3). There is no dose modification in the case of trastuzumab. In the case of a grade 3–4 toxicity trastuzumab therapy is stopped until the toxicity is reduced to grade 2 or lower.

**Table 2:** Detailed overview of the treatment protocol

Cycle	0	**1**			**2**			**3**			**4**			**5**			**6**			**7**		
**day**		**1**	**8**	**15**	**22**	**29**	**36**	**43**	**50**	**57**	**64**	**71**	**78**	**85**	**92**	**99**	**106**	**113**	**120**	**127**	**134**	**141**

history	X																					

eligibility criteria	X																					

informed consent	X																					

Trastuzumab i.v.		X^1^	X	X	X	X	X	X	X	X	X	X	X	X	X	X	X	X	X	X	X	X

Capecitabine p.o.		1–14			22–35			43–58			64–77			85–98			106–119			127–140		

vital signs, physical exam^2^	X	X	X	X	X	X	X	X	X	X	X	X	X	X	X	X	X	X	X	X	X	X

analgesic consumption^3^	X	X	X	X	X	X	X	X	X	X	X	X	X	X	X	X	X	X	X	X	X	X

pain assessment^3^	X	X	X	X	X	X	X	X	X	X	X	X	X	X	X	X	X	X	X	X	X	X

quality of life^4^	X							X						X						X		

diagnostic/remission^5^	X							X						X						X		

CA 19-9 level^6^	X	X*			X			X			X			X			X			X		

hematology^7^	X	X#	X§	X§	X	X§	X§	X	X§	X§	X	X§	X§	X	X§	X§	X	X§	X§	X	X§	X§

blood chemistry^8^	X	X			X			X			X			X			X			X		

toxicity (NCI CTC 3.0)^9^	X				X			X			X			X			X			X		

ECG	X													X								

TEE	X													X								

1. Trastuzumab: initial dose 4 mg/kg body weight, subsequent cycles 2 mg/kg body weight

2. vitals signs and physical exam: height, weight, Karnofsky performance status, pulse rate, blood pressure, temperature

3. analgesic consumption and pain symptoms (visual analogue scale)

4. EORTC QLQ-C30 (v3.0) questionnaire

5. tumor diagnostic: imaging study, HER2/neu analysis; status of remission for at least 12 weeks: CT- or MRI Abdomen, CXR (CT-chest)

6. CA19-9: serum level (ELSIA), * no measurement if between time point 0 and 1st day of cycle 1 < 4 weeks

7. Hematology: complete blood count (CBC), differential blood count; # no measurement if between time point 0 and 1st day of cycle 1 < 1 weeks; §only CBC

8. Blood chemistry: total bilirubin, ALT, AST, alk. Phos., γ-GT, total protein, albumin, creatinine, uric acid, sodium, potassium, calcium

9. NCI: CTC vers. 3.0. If toxicity > 2 reevaluation by study physical

**Table 3:** Dose modification protocol according to the level of adverse reaction caused by capecitabine therapy (Toxicity according to NCI CTC Version 3.0)

**Toxicity**	**occurrence**	**suggested measure**	**dose during following cycle (% of original dose)**
**1**		continue with current dose	100%

**2**	1st occurrence	discontinue treatment till restitution to grade 0 or 1 toxicity	100%

	2nd occurrence	discontinue treatment till restitution to grade 0 or 1 toxicity	75%

	3rd occurrence	discontinue treatment till restitution to grade 0 or 1 toxicity	50%

	4th occurrence	final discontinuation of treatment	-

**3**	1st occurrence	discontinue treatment till restitution to grade 0 or 1 toxicity	75%

	2nd occurrence	discontinue treatment till restitution to grade 0 or 1 toxicity	50%

	3rd occurrence	final discontinuation of treatment	-

**4**	1st occurrence	final discontinuation of treatment or discontinuation till restitution to grade 0 or 1 toxicity	50%

### Investigation schedule and follow-up

All patients with histologically proven pancreatic cancer undergo appropriate diagnostic studies. The radiological studies induced a chest X-ray, contrast enhanced computed tomography (CT) or magnetic resonance imaging (MRI). Laboratory tests included complete blood count, electrolytes, blood urea nitrogen, creatinine, liver function tests, bilirubin, transaminases, alkaline phosphatase, γGT, total serum protein, albumine and CA 19-9. Furthermore, to meet the inclusion criteria an electrocardiogram and a transesophageal echocardiogram is conducted by cardiologists. HER2/neu overexpression and standardized grading will be performed for each patient. Only in the case of a grade 2 overexpression additional FISH analysis will be done to assess a possible gene amplification. Patients in whom the HER2 immunohistochemistry of the tumor tissue was graded as grade 3 HER2/neu overexpression were included in the study, without performing additional FISH analysis. Also patients with the grade 2 and proven HER2 gene amplification by FISH analysis will be included in the study. To ensure a high quality of histological analysis all samples were analyzed by a highly specialized expert in pancreatic pathology (G. Klöppel, M.D.; Department of Pathology, University of Kiel, Germany). Furthermore, a thorough physical exam and measurement of vital signs will be performed prior to the start of the study as well as on day 1, 8 and 15 of each cycle. Similarly, blood tests will be performed every week and blood chemistry (including CA19-9 levels) on the beginning of each new cycle (for details see Table 2). Furthermore, since both substances have potential cardiotoxic side effects a transthoracic echocardiography is performed ever 12 weeks.

Toxicity will be evaluated using the NCI CTC (v 3.0) prior to the start of the study as well as at the beginning of each new cycle. Quality of life will be assessed using the EORTC QLQ-C30 questionnaire (v 3.0) prior to the start of the study and at the beginning of the third cycle. The assessment will then be repeated every other cycle. EORTC QLQ-C30 serves as a general measure of quality of life in cancer patients. It incorporates nine multi-item scales: five functional scales (physical, role, cognitive, emotional, and social); three symptom scales (fatigue, pain, and nausea and vomiting); and a global health and quality-of-life scale [18]. Specific symptoms (dyspnoea, insomnia, anorexia, constipation, diarrhoea, and financial impact) are measured as six single items. This instrument has been used extensively with a variety of cancer patients and was able to discriminate between individuals with metastatic and non-metastatic disease, as well as between patients at different stages of illness. The scale has good internal consistency (alpha > 0.70), and good test re-test reliability (0.80 to 0.90) [19].

In addition, analgesic consumption and assessment of pain will be documented at the beginning of each week using a visual analogue scale (VAS) for pain ranging from 1 to 10 with 10 as the highest pain score. Staging of the tumor via chest X-ray, contrast enhanced computed tomography or a magnetresonance imaging will be performed on day 43, followed by restaging every other cycle.

Patients who are no longer treated with the study medications due to tumor progression or toxicity will be followed for at least 12 months (max. 4 years).

### Statistical considerations

Primary endpoint of this study is progression free survival after 12 weeks. Secondary endpoints are listed in the section "study design and objectives". The sample size calculation is based on the two-step minimax design by Simon [20]. Trials evaluating Gemcitabine as monotherapy in advanced pancreatic cancer show a median progression free survival of 3 months, varying only little between different studies [21]. The combination of Trastuzumab and Capecitabine would be considered ineffective if the median progression-free survival after 3 months would be 50% or less. The combination therapy would, on the other hand, be deemed effective if the progression-free survival would be 70% or more. Assuming an accrual period of 36 months and a follow-up period of 12 to 48 months, testing for a difference in hazard on level α = 0.05 with a power of 80% a study sample size of 37 patients is needed. After 23 patients an interim analysis will be carried out.

According to the "intention-to-treat" principle all patients fulfilling the inclusion- and exclusion criteria and who have started chemotherapy are included in the evaluation, i.e. even patients who show a fast tumor progression, who die before tumor progression can be evaluated or who die due to treatment related toxicities are included. In addition, "per protocol" analysis of the primary end point will be performed. For this purpose only patients who have received at least 2 cycles of the combination therapy are included. Furthermore, all patients who have received at least one cycle of the combination therapy are evaluated for toxicities and side-effects.

If less than 13 of the 23 patients during the interim analysis show progression-free survival the combination therapy might be considered ineffective and the study could be stopped. The degree of HER2 expression of the included patients will be taken into account before the trial is stopped. If, on the other hand, 13 or more of the 23 patients during interim analysis exhibit progression-free survival the recruitment will be extended to 37 patients.

The progression free survival after 3 months will be calculated with a confidence interval of 95%. Progression free survival and overall survival will be summarized by Kaplan and Meier estimate. The probability of remission (complete or partial) over time will be calculated using cumulative incidence rates. The duration of remission will be summarized by Kaplan and Meier estimates. The clinical benefit response (CBR) probability 12 weeks after initiation of therapy will be estimated by the number of patients that exhibit a CBR within 12 weeks. The correlation between the CA19-9 and HER2 levels with progression free survival time will be analyzed using Cox's regression model. The rate of adverse events or severe adverse events will be given using a 95% confidence interval.

## Discussion

Most patients with pancreatic cancer will have to be treated by chemotherapy. So far only limited success has been achieved with this kind of treatment. Addition of multimodal therapies, including radiation and chemotherapy regimes has had only minimal effect on the prognosis of these patients [22,23]. Up to now the standard treatment for these patients is chemotherapy with gemcitabine which has been shown to be marginally more effective than 5-FU in alleviation of disease-related symptoms [3]. Nevertheless the overall prognosis of these patients is dismal [2]. Therefore, new treatment strategies including new targeted therapies are necessary to improve the overall prognosis for patients with pancreatic cancer.

The anti-HER2 antibody trastuzumab is a promising new candidate in the treatment of advanced pancreatic adenocarcinoma, because HER2 is overexpressed in a subset of ductal adenocarcinoms of the pancreas [16]. Furthermore, Trastuzumab has already shown its therapeutic potential in combination with other chemotherapeutic drugs in the treatment of HER2 overexpressing breast cancer [12]. In addition a number of preclinical studies and pilot studies in humans have shown the effectiveness of Trastuzumab in HER2 overexpressing pancreatic adenocarcinoma [13-15]. Because trastuzumab is generally well tolerated and side effects are rare this compound is an ideal new candidate for the treatment of pancreatic cancer and there is a clear interest in evaluating the clinical efficacy of this new, highly specific compounds [24,25].

This study is a prospective, non-randomized multi-centered Phase II clinical trial evaluating the clinical effects of Capecitabine in combination with the monoclonal anti-HER2 antibody Trastuzumab in patients with non-resectable, HER2 overexpressing pancreatic cancer. Apart from the oncological efficacy of this treatment combination, its toxicity as well as the quality of life of this therapeutic regimen will be assessed. Furthermore, the trial should provide evidence for the relationship between progression free survival and CA19-9 plasma levels as a predictive therapeutic factor. The results of this study will definitely contribute to our current clinical and scientific knowledge on the treatment of locally advanced pancreatic adenocarcinoma.

## Competing interests

Roche Pharma AG, Grenzach, Germany has supplied the study medication and has paid for registration of this trial at the International Standard Randomized Controlled Trial Number Register (ISRCTN).

## Authors' contributions

All authors substantially contributed to the current manuscript as listed below:

AM drafted the manuscript. PB revised the manuscript, JH, RH, MG, SK, WS, VH, EE and TS were involved in the conception and acquisition of data of this study. GK performed the pathological analysis of specimens. MG is the principal investigator of the study and designed this clinical trial.

## Pre-publication history

The pre-publication history for this paper can be accessed here:



## References

[B1] Jemal A, Siegel R, Ward E, Hao Y, Xu J, Murray T, Thun MJ (2008). Cancer statistics, 2008. CA Cancer J Clin.

[B2] Fesinmeyer MD, Austin MA, Li CI, De Roos AJ, Bowen DJ (2005). Differences in survival by histologic type of pancreatic cancer. Cancer Epidemiol Biomarkers Prev.

[B3] Burris HA, Moore MJ, Andersen J, Green MR, Rothenberg ML, Modiano MR, Cripps MC, Portenoy RK, Storniolo AM, Tarassoff P, Nelson R, Dorr FA, Stephens CD, Von Hoff DD (1997). Improvements in survival and clinical benefit with gemcitabine as first-line therapy for patients with advanced pancreas cancer: a randomized trial. J Clin Oncol.

[B4] Sultana A, Smith CT, Cunningham D, Starling N, Neoptolemos JP, Ghaneh P (2007). Meta-analyses of chemotherapy for locally advanced and metastatic pancreatic cancer. J Clin Oncol.

[B5] Reigner B, Blesch K, Weidekamm E (2001). Clinical pharmacokinetics of capecitabine. Clin Pharmacokinet.

[B6] Cartwright TH, Cohn A, Varkey JA, Chen YM, Szatrowski TP, Cox JV, Schulz JJ (2002). Phase II study of oral capecitabine in patients with advanced or metastatic pancreatic cancer. J Clin Oncol.

[B7] Hess V, Salzberg M, Borner M, Morant R, Roth AD, Ludwig C, Herrmann R (2003). Combining capecitabine and gemcitabine in patients with advanced pancreatic carcinoma: a phase I/II trial. J Clin Oncol.

[B8] Jensen SA, Sorensen JB (2006). Risk factors and prevention of cardiotoxicity induced by 5-fluorouracil or capecitabine. Cancer Chemother Pharmacol.

[B9] Herrmann R, Bodoky G, Ruhstaller T, Glimelius B, Bajetta E, Schuller J, Saletti P, Bauer J, Figer A, Pestalozzi B, Kohne CH, Mingrone W, Stemmer SM, Tamas K, Kornek GV, Koeberle D, Cina S, Bernhard J, Dietrich D, Scheithauer W (2007). Gemcitabine plus capecitabine compared with gemcitabine alone in advanced pancreatic cancer: a randomized, multicenter, phase III trial of the Swiss Group for Clinical Cancer Research and the Central European Cooperative Oncology Group. J Clin Oncol.

[B10] Bernhard J, Dietrich D, Scheithauer W, Gerber D, Bodoky G, Ruhstaller T, Glimelius B, Bajetta E, Schuller J, Saletti P, Bauer J, Figer A, Pestalozzi BC, Kohne CH, Mingrone W, Stemmer SM, Tamas K, Kornek GV, Koeberle D, Herrmann R (2008). Clinical benefit and quality of life in patients with advanced pancreatic cancer receiving gemcitabine plus capecitabine versus gemcitabine alone: a randomized multicenter phase III clinical trial – SAKK 44/00-CECOG/PAN.1.3.001. J Clin Oncol.

[B11] Boeck S, Hoehler T, Seipelt G, Mahlberg R, Wein A, Hochhaus A, Boeck HP, Schmid B, Kettner E, Stauch M, Lordick F, Ko Y, Geissler M, Schoppmeyer K, Kojouharoff G, Golf A, Neugebauer S, Heinemann V (2008). Capecitabine plus oxaliplatin (CapOx) versus capecitabine plus gemcitabine (CapGem) versus gemcitabine plus oxaliplatin (mGemOx): final results of a multicenter randomized phase II trial in advanced pancreatic cancer. Ann Oncol.

[B12] Pegram MD, Konecny GE, O'Callaghan C, Beryt M, Pietras R, Slamon DJ (2004). Rational combinations of trastuzumab with chemotherapeutic drugs used in the treatment of breast cancer. J Natl Cancer Inst.

[B13] Buchler P, Reber HA, Buchler MC, Roth MA, Buchler MW, Friess H, Isacoff WH, Hines OJ (2001). Therapy for pancreatic cancer with a recombinant humanized anti-HER2 antibody (herceptin). J Gastrointest Surg.

[B14] Kimura K, Sawada T, Komatsu M, Inoue M, Muguruma K, Nishihara T, Yamashita Y, Yamada N, Ohira M, Hirakawa K (2006). Antitumor effect of trastuzumab for pancreatic cancer with high HER-2 expression and enhancement of effect by combined therapy with gemcitabine. Clin Cancer Res.

[B15] Buchler P, Reber HA, Eibl G, Roth MA, Buchler MW, Friess H, Isacoff WH, Hines OJ (2005). Combination therapy for advanced pancreatic cancer using Herceptin plus chemotherapy. Int J Oncol.

[B16] Safran H, Steinhoff M, Mangray S, Rathore R, King TC, Chai L, Berzein K, Moore T, Iannitti D, Reiss P, Pasquariello T, Akerman P, Quirk D, Mass R, Goldstein L, Tantravahi U (2001). Overexpression of the HER-2/neu oncogene in pancreatic adenocarcinoma. Am J Clin Oncol.

[B17] Safran H, Iannitti D, Ramanathan R, Schwartz JD, Steinhoff M, Nauman C, Hesketh P, Rathore R, Wolff R, Tantravahi U, Hughes TM, Maia C, Pasquariello T, Goldstein L, King T, Tsai JY, Kennedy T (2004). Herceptin and gemcitabine for metastatic pancreatic cancers that overexpress HER-2/neu. Cancer Invest.

[B18] Aaronson NK, Ahmedzai S, Bergman B, Bullinger M, Cull A, Duez NJ, Filiberti A, Flechtner H, Fleishman SB, de Haes JC (1993). The European Organization for Research and Treatment of Cancer QLQ-C30: a quality-of-life instrument for use in international clinical trials in oncology. J Natl Cancer Inst.

[B19] Hjermstad MJ, Fossa SD, Bjordal K, Kaasa S (1995). Test/retest study of the European Organization for Research and Treatment of Cancer Core Quality-of-Life Questionnaire. J Clin Oncol.

[B20] Simon R (1989). Optimal two-stage designs for phase II clinical trials. Control Clin Trials.

[B21] Heinemann V (2002). Gemcitabine in the treatment of advanced pancreatic cancer: a comparative analysis of randomized trials. Semin Oncol.

[B22] Regine WF, Winter KA, Abrams RA, Safran H, Hoffman JP, Konski A, Benson AB, Macdonald JS, Kudrimoti MR, Fromm ML, Haddock MG, Schaefer P, Willett CG, Rich TA (2008). Fluorouracil vs gemcitabine chemotherapy before and after fluorouracil-based chemoradiation following resection of pancreatic adenocarcinoma: a randomized controlled trial. JAMA.

[B23] Neoptolemos JP, Stocken DD, Friess H, Bassi C, Dunn JA, Hickey H, Beger H, Fernandez-Cruz L, Dervenis C, Lacaine F, Falconi M, Pederzoli P, Pap A, Spooner D, Kerr DJ, Buchler MW (2004). A randomized trial of chemoradiotherapy and chemotherapy after resection of pancreatic cancer. N Engl J Med.

[B24] El Fitori J, Su Y, Buchler P, Ludwig R, Giese NA, Buchler MW, Quentmeier H, Hines OJ, Herr I, Friess H (2007). PKC 412 small-molecule tyrosine kinase inhibitor: single-compound therapy for pancreatic cancer. Cancer.

[B25] Buchler P, Reber HA, Roth MM, Shiroishi M, Friess H, Hines OJ (2007). Target therapy using a small molecule inhibitor against angiogenic receptors in pancreatic cancer. Neoplasia.

